# Vitamin Status in Children with Cystic Fibrosis Transmembrane Conductance Regulator Gene Mutation

**DOI:** 10.3390/nu14214661

**Published:** 2022-11-04

**Authors:** Paulina Wysocka-Wojakiewicz, Halina Woś, Tomasz Wielkoszyński, Aleksandra Pyziak-Skupień, Urszula Grzybowska-Chlebowczyk

**Affiliations:** 1 Department of Pediatrics, Faculty of Medical Sciences, Medical University of Silesia, 40-752 Katowice, Poland; 2 Faculty of Health Sciences, University of Bielsko-Biala, 43-309 Bielsko-Biała, Poland; 3 Higher School of Strategic Planning and Laboratory Medicine Centre, 41-303 Dąbrowa Górnicza, Poland; 4 Department of Children’s Diabetology, Silesian Medical University in Katowice, 40-752 Katowice, Poland

**Keywords:** vitamin A, D, E, and C, CFSPID, cystic fibrosis, inconclusive diagnosis

## Abstract

Background: The issue of vitamin metabolism in children with cystic fibrosis screen positive, inconclusive diagnosis (CFSPID) is not well known. The aim of this study was to determine the status of vitamins A, D, E, and C in the blood of a group of children with CFSPID. Material and Methods: A total of 89 children were enrolled in the study (Me: 3.6 years, 52.8% boys), as follows: 28 with CFSPID, 31 with CF (cystic fibrosis), and 30 HC (healthy children). Their blood concentrations of vitamins A, D, E, and C, and their dietary intake of these vitamins were analysed in the study groups on the basis of a three-day food diary. Results: The patients with CFSPID had significantly higher serum vitamin D (*p* = 0.01) and E (*p* = 0.04) concentrations, compared to the children with CF. None of the children with CFSPID revealed vitamin A or E deficiencies. Patients with CF had been consuming significantly higher vitamin D and E amounts (*p* = 0.01). The vitamin concentrations did not depend either on the pancreatic/liver function or on anthropometric parameters. In total, 32.14% of patients with CF did not cover the baseline recommended calorie intake, and 53.6% and 36% did not take the recommended vitamin E and vitamin A intake, respectively. Conclusion: Children with CF and CFSPID did not fully cover the dietary recommendations for vitamin supply, but vitamin deficiency was found only in CF.

## 1. Introduction

Cystic fibrosis (CF) is the most common autosomal, recessively inherited monogenic disease in the Caucasian population. It is estimated that the prevalence of the disease in Poland is 1:4394–5000 of live births [1]. So far, more than 2000 mutations of the CFTR gene have been detected, which can be divided into six classes, depending on the molecular abnormality, with some mutations belonging to more than one class [2,3]. In 2008, a classification was proposed, taking into account the clinical significance of the mutations [4,5]. The screening test model for newborn screening (CF NBS) has been changing over the years. Since 2011, the IRT (immunoreactive trypsinogen/trypsin)/DNA/EGA (extended gene analysis) model, which allows the detection of 95% of mutant alleles in the Polish population, has been implemented in Poland. Based on an extended DNA analysis, we can detect not only cystic fibrosis patients but also mutated gene carriers, patients with CFTR-dependent diseases, and children with CFSPID.

The definition of CFSPID (cystic fibrosis screen positive, inconclusive diagnosis) includes children with a positive neonatal screening, in whom cystic fibrosis cannot be unequivocally confirmed due to the following: the absence of symptoms of the disease; the presence of the CFTR gene mutation that is of, as yet, unknown clinical significance; and/or an abnormal sweat test result, but less than 60 mmol/L. Children with CFSPID constitute a challenge for CF specialists. The natural history of CFSPID is still unclear. There are no standardized protocols or predictors of reclassification from CFSPID to CF or a CFTR-related disorder. The increase in sweat chloride concentration with age may be associated with a risk of reclassification [6]. There is clear evidence that infants with an intermediate sweat chloride value are more likely to convert to a CF diagnosis through a rise in subsequent sweat values or clinical features [7]. The reported rate of conversion or reclassification from a CRMS (cystic fibrosis transmembrane conductance regulator-related metabolic syndrome)/CFSPID designation to a CF diagnosis varies from 6% to 48% [8].

It is commonly known that there is a deficiency of fat-soluble vitamins in the group of children with cystic fibrosis. Fat-soluble vitamin deficiency is present even in 10–35% of children with CF and pancreatic insufficiency [9]. The issue of vitamin metabolism in children with cystic fibrosis screen positive, inconclusive diagnosis (CFSPID) is not well known. To our knowledge, none of the prior studies have comprehensively assessed the vitamin and nutritional status of children with CFSPID. So far, the diet and the implementation of nutritional recommendations in this group of children have not been analysed. Prognostic markers to determine the risk of reclassification from CFSPID to CF are still being researched. The early diagnosis of fat-soluble vitamin deficiency in this group of children would encourage the modification of dietary recommendations and the implementation of treatments earlier to prevent long-term complications. Currently, children with CFSPID are only followed up.

The aim of this study was to determine the status of vitamins A, D, E, and C in the blood of a group of children with CFSPID, in relation to children with CF and to healthy controls, and to analyse the relationship of those vitamins with selected markers of the pancreatic and liver functions of the studied patients.

## 2. Materials and Methods

A total of 89 children, aged 2 months to 17 years, were included in the analysis (Me (median) = 3.6 years.; the IQR (interquartile range) was 1.06–7.64; and 52.8% were boys). They were treated at the department of paediatrics and at the outpatient clinic for the treatment of cystic fibrosis, of the Upper Silesian Children’s Medical Centre, in Katowice, Poland. The patients were divided into the following 3 groups: CFSPID (*n* = 28), CF (*n* = 31), and a control group—healthy children (*n* = 30). The group with CFSPID consisted of children with a positive CF NBS result, with two mutations of the CFTR gene, including at least one of which had unclear phenotypic consequences. Those children who did not meet the diagnostic criteria for CF, who were not chronically ill, and who had acute infections were excluded. The CF group included children with 2 CF-causing mutations in both alleles of the CFTR gene, confirmed by sweat test. Those children were in a good clinical condition, without exacerbations during the previous 3 months and without other concomitant diseases or *Peudomonas aeruginosa* colonisation.

In the patients with CFSPID and CF, several assays were carried out, including faecal elastase (by ELISA), serum pancreatic lipase, ALT, AST, GGT, bile acids, and total cholesterol level. The cholesterol concentration was used as an input to calculate a standardised serum vitamin E concentration, as α-tocopherol/cholesterol ratio, which should, ultimately, exceed 5.4 mg/g in paediatric patients with CF [10]. Biochemical tests were performed by standard methods. In addition, vitamin A, D, E, and C concentrations were determined in all the 3 study groups. The concentrations of vitamins A and E, vitamin C, and 25(OH)D were determined by HPLC, the kinetic-spectrophotometric method [11], and the chemiluminescent method, respectively (see the range of standards in Appendix A). A fasting blood sample was collected in the morning. Faecal elastase was measured at the time of admission to the study. 

Children’s dietary history was also analysed from the last 3 days before the samples for laboratory tests were collected and vitamin supplementation was assessed during the previous 3 months before inclusion into the study. Portion size and the weight of consumed meals and snacks were taken into account. Both the calorie content of the meals and the vitamin A, D, E, and C content in the diet were calculated. In the CFSPID group, energy requirements were assumed as for healthy peers with moderate physical activity and on the basis of dietary standards for the Polish population. The recommended daily allowance (RDA) for vitamins A, E, and C, and the adequate intake (AI) for vitamin D and infant vitamins were considered [12,13]. In the CF group, the percentage of use of the daily energy requirement, meeting the recommendations for CF patients, was considered, and was taken as 120% of the recommended energy supply vs. healthy peers [14]. No dietary history was obtained from 3 CF patients. A programme from the kcalmar.com platform was used to calculate the nutrient content of the diet.

Anthropometric measurements (body weight and body height) were taken in line with the current measurement techniques. Based on the following data: age, sex, weight, and height, the BMI z-score and z-scores for weight and height of the subjects were calculated. According to WHO, weight deficiency was diagnosed, when the BMI z-score was ≤−2 SD, SD values ≥ 1 were taken as borderline for overweight, and SD ≥ 2 for obesity. A similar reference range of z-score values was adopted for weight and height [15].

The authors analysed the presence and type of mutations in the CFTR gene in children with CFSPID and CF, taking into account the clinical significance of the mutation and the co-occurrence of the F508del mutation (homo-, heterozygotes, and other/other).

Statistical analysis: Quantitative variables were presented as median and interquartile range (IQR) values, and qualitative variables were presented by means of absolute values and percentages. The normality of distribution was verified using the Shapiro–Wilk test. When comparing the differences in the assessed parameters between the study groups, in case of the normal distribution of numerical data, Student’s *t*-test was used and, in cases when distribution deviated from normal, the analysis was performed using the non-parametric Mann–Whitney test. Comparing the differences in the assessed parameters between more than two study groups, in the case of normal distribution, one-way analysis of variance was used. For significant variables, the Bonferoni test was used, and with a distribution that was different from normal—the analysis was carried out using the non-parametric test. For statistical evaluation of differences in the frequency of the analysed characteristics, the chi2 test (with or without Yates’ correction) or Fisher’s exact test was used, depending on the size of the groups. For correlation analysis, depending on the distribution and type of variables, the Spearman correlation test or the Pearson correlation test was used. A *p* value < 0.05 was accepted as the threshold for statistical significance. The study was approved by the Biotics Committee of the Silesian Medical University in Katowice, Resolution No. KNW/0022/KB1/9/I/16, of 06.06.2016.

## 3. Results

### 3.1. Characteristics of the Groups

The study groups showed no statistically significant differences in terms of age and gender (*p* > 0.05). See Table 1 for the nutritional status data of the individual subjects. No significant correlation was observed among the concentrations of vitamins A, D, E, and C, or between the gender and the z-score of the weight, height, and BMI (*p* > 0.05) of the participants.

### 3.2. Results of Laboratory Tests

In all the children with CFSPID (100%), the faecal elastase (FE-1) concentrations were within their normal limits of >500 μg/g of faeces. In the CF group, 29 (93.54%) children had abnormal elastase levels—<200 μg/g of stools—which were indicative of pancreatic exocrine dysfunctions. In two children with CF, the faecal elastase concentrations were >200 μg/g of faeces. These were girls, aged three and seven years, with CFTR gene mutations, G542X/3272-26A→G and F508del/3272-26A→G, respectively. The other results were within the reference norms or slightly above the age limits. This was not relevant to the study. Neither in the CFSPID nor in the CF groups was there any significant correlation observed between pancreatic and liver function exponents and the blood levels of vitamins A, D, E, and C (*p* > 0.05)

### 3.3. Vitamins

In most of the children, vitamin A, D, E, and C concentrations remained in the normal range. None of the children with CFSPID showed any significant deficiency of fat-soluble vitamins, and only two children demonstrated suboptimal levels of vitamin D. Regarding vitamin D, the vast majority of the subjects with CFSPID and the healthy children had optimal vitamin D levels (30–50 ng/mL). Most deficient (<20 ng/mL) and suboptimal vitamin D concentrations (20–30 ng/mL) were observed among the children with CF, in 5 (16.1%) and 14 (45.2%), respectively. The differences were statistically significant (*p* = 0.001). No vitamin A excess was noted in any of the children. Vitamin E deficiency in the study group was found in only two (6.5%) children with CF (siblings); however, taking into account the α-tocopherol/cholesterol ratio, the percentage was higher and amounted to seven (22.6%). The vitamin A, D, E, and C status among the children in the study groups is shown in Table 2. 

Table 3 shows the distribution of the blood concentrations of particular vitamins.

Children with cystic fibrosis had significantly lower serum levels of vitamin D and E (although within the normal range), compared to those with CFSPID and the healthy patients, despite supplementation.

**Figure 1:**
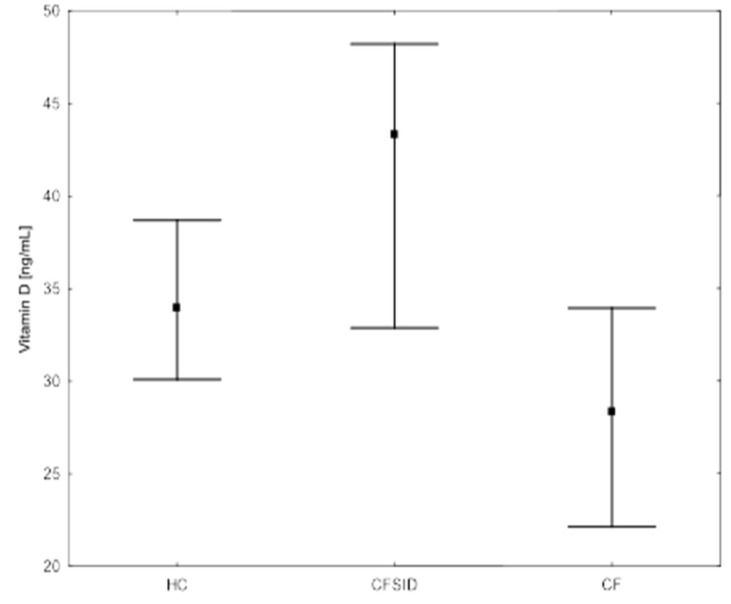
Vitamin D blood concentration among CF, CFSPID, and HC.

**Figure 2:**
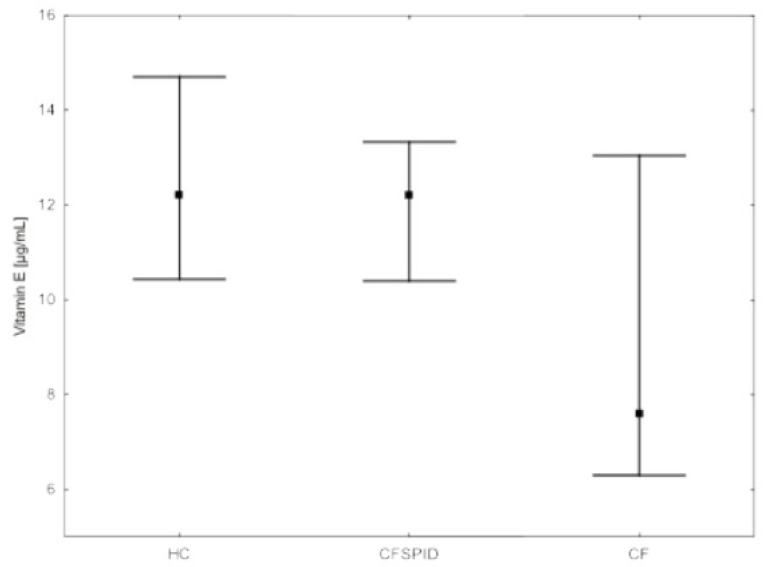
Vitamin E blood concentration among CF, CFSPID, and HC.

Considering the corrected α-tocopherol/cholesterol concentration, the values of <5.4 mg/g were observed in seven (22.6%) CF patients, whereas in all the CFSPID children the ratio was >5.4 mg/g. The vitamin D and vitamin E blood concentration among the CF, CFSPID and HC, the median and interquartile ranges are presented in Figure 1 and Figure 2, respectively.

### 3.4. Dependence of Serum 25(OH)D Metabolite Concentrations on the Season of the Year

The majority of patients had their blood samples taken during the months of May to October (23 healthy subjects, 22 children with CFSPID, and 21 patients with CF). The differences in the frequency of the blood sample collection by season were not statistically significant (*p* = 0.591). The lowest 25(OH)D metabolite concentrations were observed in the children with blood samples taken during the winter months (*p* = 0.024).

### 3.5. Vitamins in Diet

An analysis of a three-day dietary diary of the CFSPID and CF patients showed the distribution of vitamins A, D, E, and C in their diets (see Table 4). The patients with CF consumed significantly more dietary vitamin D and E (*p* = 0.001). The dietary vitamin intake and supplementation had no significant effect on the assayed vitamin concentrations in their blood (*p* > 0.05). The data are presented in Table 5.

### 3.6. Adherence to Dietary Recommendations, Regarding Vitamin Intake

For the children with CFSPID, their dietary vitamin intake standards were established based on the estimated average requirement (EAR), the recommended dietary allowance (RDA), and the adequate intake (AI) for the healthy children in the Polish population [12]. These standards can be found in the Appendix A. For the children with CF, the 2002 guidelines were used for the statistical calculations regarding the children with CF, according to Borowitz et al. [16].

The adherence to the recommendations for the daily vitamin intake in the CF and CFSPID groups is shown in Table 6. No child with CFSPID was significantly deficient in vitamin A, D or E, although not all the children met their daily requirements for those vitamins. The differences in the total vitamin intake between the CFSPID and CF groups were significant only for vitamins D and E (*p* = 0.001).

### 3.7. Adherence to Dietary Recommendations in CFSPID and CF Groups

European guidelines recommend that energy intake for people with CF range from 120% to 150% of the energy needs of the healthy population of a similar age, sex, and size [17]. Among the children with CFSPID, 46.5% did not meet their daily caloric requirements for healthy children, with moderate physical activity. The vast majority of children with CF did not take in the recommended number of calories per day during the study period. In total, 89.28% of patients did not meet 120% of the energy requirements of their healthy peers, with moderate physical activity. In total, 32.14% of the children with CF did not meet the baseline recommended caloric intake, yet they did not differ significantly in anthropometric parameters from the healthy children and those with CFSPID. Those patients periodically benefited from industrial diet support, but not during this study. A comparison of the caloric supply between the CFSPID and CF groups is presented in Table 7. The CF children consumed more calories, but the supply did not differ significantly from the caloric content in the diet of the children with CFSPID.

### 3.8. Correlations in the Groups

When comparing the patients in the CFSPID group with the HC, no statistically significant differences were found in the concentration of vitamins and other laboratory parameters, apart from higher 25(OH)D levels in the children with CFSPID (*p* = 0.043). Children with CFSPID had a statistically significantly higher concentration of vitamin E, vitamin D, cholesterol, and lipase, and they had a higher α-tocopherol/cholesterol ratio compared to the CF patients (*p* < 0.05). All the collected clinical and laboratory parameters in the CFSPID group were analysed with the concentration of the determined vitamins. A statistically significant negative correlation was observed between vitamin A and GGTP (−0.641, *p* = 0.018), and between vitamin D and age (*R* = −0.464, *p* = 0.015). The other correlations were statistically insignificant (*p* > 0.05). A similar analysis was performed in the CF group. There was a positive correlation between vitamin A and cholesterol (*R* = 0.409, *p* = 0.022); a positive correlation between vitamin E, lipase (*R* = 0.373, *p* = 0.039), and cholesterol (*R* = 0.495, *p* = 0.005); a negative correlation between vitamin E and ALT (*R* = −0.417, *p* = 0.022); a positive correlation between vitamin D and body height (*R* = 0.916, *p* = 0.020); and the other correlations were not significant statistically (*p* > 0.05).

## 4. Discussion

In our study, neither the CF nor the CFSPID children were significantly different with respect to the healthy children in terms of their anthropometric measurements. In the group of patients with CF, this was probably due to the early enzyme replacement therapy, and to the adequate diet and vitamin supplementation. On the other hand, in the group of children with CFSPID, this resulted from higher parental awareness, a better care for a healthy lifestyle, and the absence of any diagnosed metabolic disorders. Despite the application of specific dietary recommendations, the deficiency of assayed fat-soluble vitamins mainly affected the children with CF.

### 4.1. Vitamin A

The determination of an optimal vitamin A supplementation dose for children with CF is still a problematic issue. Only since 2016, on the basis of ESPGHAN guidelines, it has been recommended that we supplement retinol in low doses, increasing them under serum concentration control until normal values are reached [17]. Previously, the supplemented dose depended on a child’s age, so both vitamin A deficiency and excess were often found. A vitamin A deficiency among the CF patients (adults and children) in Poland was described in approximately 16% of them, and, in Australia, vitamin A deficiency was described in 11% of the examined children [9,18]. The results of our study are consistent with the reports of these authors: in our study, we found vitamin A deficiency in almost 10% of the examined children with CF. By contrast, in other publications prior to the recommendation change, almost no vitamin A deficiency was observed in children and adults with CF, while vitamin A excess was more often identified [19,20,21]. Due to the differences in the supplemented retinol doses, a direct comparison of those results is rather unfeasible. 

In our study, we did not confirm any relationship between vitamin A concentrations and nutritional status, diet, or supplementation. Neither did vitamin A concentrations depend on pancreatic exocrine function or liver function. These results were consistent with the reports of other authors [18,19,21,22]. By contrast, in the study by Maqbool et al., retinol concentrations inversely correlated with standardised weight and height [21]; however, in the aforementioned study, the CF patients differed significantly in weight and height, while the age range of CF subjects also included adults up to 25 years of age.

In our study, most of the children followed the dietary recommendations for vitamin A intake, either in diet or in supplements, and a significant deficit of that vitamin was observed only in the group of children with CF. No one was found to have hypervitaminosis A. In the studies by Brei, Maqbool, and Graham-Maar, the total supply of vitamin A with food and vitamin preparations was much higher than recommended, resulting in elevated and even toxic serum vitamin A levels in some patients [20,21,23]. Nowadays, thanks to the change in the guidelines and the recommended annual monitoring of serum retinol levels, the toxic effects of vitamin A are no longer observed in children with CF. 

### 4.2. Vitamin D

The guidelines of Polish scientific societies recommend a year-round vitamin D supplementation when skin synthesis is insufficient, especially between September and April. Depending on latitude, the severity of vitamin D deficiency among the studied children with CF varies, ranging from 24% in the French study by Munck, to 90% in the US study by Rovner, AJ [24,25]. Our results are close to the Polish results of Sands’ study [26], where 25(OH)D concentrations <30 ng/mL in children with CF were found in 79% of the group and, in our study, in 61% of the children with CF. Munck’s publication first investigated vitamin D concentrations in childrenwith CFSPID, finding a deficit in 18% of the subjects [24]. In contrast, in our study, the children with CFSPID had significantly higher serum 25(OH)D concentrations than the other subjects, and suboptimal concentrations were found in only 7.4%. This was mainly due to parental care, adequate sun exposure, and proper supplementation.

Seasonal variability in vitamin D concentrations and an inverse relationship of vitamin D concentrations to age were observed both in our study and in previous publications [27,28,29,30,31]. In our study, we did not confirm any correlation between vitamin D levels and the nutritional status. This was because the tested groups did not differ significantly in that particular parameter. We did not confirm any association among vitamin D concentrations, and exocrine pancreatic and hepatic function. These results were consistent with the reports of other authors [29,32,33]. Data on the correlation of vitamin D with other fat-soluble vitamins are rather inconclusive. In our study, we demonstrated a positive correlation between vitamin D and vitamin E, as did Grey et al. [34], while other authors did not confirm it [29,33].

In our study, all the patients with CF consumed the recommended amount of vitamin D, yet most of them were found to be deficient in that vitamin. Compared to the synthesis in the skin, the diet covers a maximum of 20% of the daily vitamin D requirement [35], therefore, the analysis of the total dietary intake of vitamin D was not sufficient, either for CF patients or for the other groups. In Munck’s study, a deficiency of vitamin D occurred in 24% of the patients in the cohort with CF—all receiving vitamin supplementation—and in 18% of the inconclusive CF cohort, 60% of whom were receiving a half dosage of fat-soluble vitamins [24]. There are few recommendations for children with CFSPID, so these children should consume/supplement vitamin D in doses appropriate for healthy children, taking into account the duration of their exposure to sun light.

Similarly, as in Rovner’s study, we did not prove any relationship between the dietary vitamin D content and the serum levels in any of the studied groups [25]. Different results were obtained by Timmers, but he used the criterion of dividing the ingested dose of vitamin D kg/body weight [30]. Serum 25(OH)D concentrations also depend on the variation of the genes involved in vitamin D metabolism. In planning further studies on the pleiotropic effect of vitamin D, all the previously mentioned variables should be taken into account.

### 4.3. Vitamin E

Since α-tocopherol binds to lipids, in order to assess serum vitamin E levels, it seems more appropriate to calculate the ratio of α -tocopherol to total blood lipids or to cholesterol, which should, ultimately, exceed 5.4 mg/g in paediatric patients with CF [10,36]. This should be taken into account, especially among patients with liver disease and adiposity disorders [37]. In our study, vitamin E deficiency was found in 6.5% of preschool-aged children with CF (siblings) only, whereas, taking into account the α-tocopherol/cholesterol ratio, the percentage was higher—22.6%and mainly concerned the school-aged children. Most authors agree that the prevalence of vitamin E deficiency in CF patients depends on the age of the subjects and mainly affects older patients [9]. Sapiejka’s results coincide with ours. α-tocopherol deficiency was found in 8.0% of subjects and in the group of children at 12–17 years, in 14.8% [38]. Recently, hypervitaminosis E has been more common than vitamin E deficiency. In our study, an excess of vitamin E was found in each of the study groups and in a similar proportion, as follows: CF (6.5%), CFSPID (10.7%), and healthy children (10.0%). These data are comparable to Sapiejka and Woestenenk’s results in CF patients, being 11.4% and 12%, respectively [38,39]. This can be explained by Woestenenk’s hypothesis that vitamin E deficiency is more related to chronic inflammation and exacerbations of lung disease than to dietary deficiencies [39]. Children with CFSPID and HC, despite their lack of supplementation for vitamin E, showed significantly higher serum α-tocopherol levels vs. the CF patients (although 89% were within the normal range).

In our study, we did not confirm the relationship between vitamin E concentrations and nutritional status, diet, supplementation, or liver function, thus, our results were consistent with the publications by other authors [10,38,40,41]. The results regarding the correlation of vitamin E concentrations with pancreatic exocrine function, demonstrate the highest differentiation. In our study, we did not confirm the relationship, either based on faecal elastase −1 or serum lipase levels, as did other authors [22,42]; however, some publications have documented such an association [9,40].

Both in our study and in Woestenenk’s publication, the supply of vitamin E in supplements was higher than in the diet of the patients with CF. Although vitamin E intake among children with CF did not meet the dietary recommendations for CF, a α-tocopherol deficiency was rarely found [39]. In the presented material, the children with CF consumed significantly more dietary vitamin E than the children with CFSPID, and α-tocopherol deficiency was found only among the children with CF. By contrast, in Huang et al.’s study, most children with CF followed the CF Foundation guidelines regarding the volume of supplementation doses, demonstrating higher α-tocopherol concentrations in their blood serum and a higher α-tocopherol/cholesterol ratio than the children in a control group [10]. These differences resulted from the division of the obtained results into percentiles of the normal range, which was considered in other studies.

### 4.4. Vitamin C

There are few reports discussing the issue of water-soluble vitamins in children with CF and CFSPID. Most publications report no problems with vitamin C deficiency in CF patients [43]. According to the ESPEN-ESPGHAN-ECFS guidelines, vitamin C should be supplemented only in the case of its deficiency [17]. Among our patients, plasma vitamin C deficiency and excess were found in each of the study groups. In our study, we did not confirm any correlation between vitamin C levels and the nutritional status, or the pancreatic and liver function exponents, which was consistent with the reports by Brown RK and Winklhofer-Roob [44,45]. The results of previous analyses have shown that vitamin C intake covers the dietary standards for the groups studied in Poland [46,47]. In our study, three children in the CFSPID and one child in the CF group did not meet their vitamin C requirements. This was not reflected in their plasma vitamin C concentrations and was due to reduced fruit and vegetable intake vs. their peers. In our study, almost all the children with CF consumed the recommended vitamin C intake. Similar results were reported in Back’s study, where the recommendations for vitamin C intake were met by 95% of the subjects [43]. Although the deficiency of water-soluble vitamins is a marginal problem among CF patients, it is still present and, thus, requires further research on the prevalence of this phenomenon in larger numbers of patients.

### 4.5. Diet Analysis of Children with CFSPID and CF

Our study also assessed adherence to dietary recommendations for energy intake and dietary vitamin supplementation among CF and CFSPID patients. An analysis of a three-day food diary showed that 46.5% of CFSPID and 32.14% of CF patients had not met the recommended daily energy requirements (RDA), respectively.

Those patients periodically benefited from industrial diet support, but not during the study. Nevertheless, the nutritional status of the studied children with cystic fibrosis was not significantly different from the other groups. This is, in general, possible by an early detection of the disease in neonatal screening and by the application of early nutritional interventions and enzyme replacement therapy. In the Calvo-Lerma study, up to 46% of children with CF did not take the standard daily dietary energy supply [48]. In our study, in the group of children with CF, the recommendations for the intake of vitamins E and A were met by only 46.4% and 64.3% of the patients, respectively, and the recommended intake of vitamins D and C were also not met by the vast majority of patients, amounting to 90% and more. In the group of children with CFSPID, the recommended amount of vitamin D in the diet was consumed by only 10.7%, while vitamin E was consumed by 60.7% of the subjects, and vitamins A and C were also consumed by almost 90% of the patients. The total vitamin intake in the group of patients with cystic fibrosis had no such significant effects on serum vitamin concentrations, as they did among the children with CFSPID. There are few publications that have reported on the adherence to dietary recommendations in a group of children with CFSPID.

It is important to draw the attention of children with CF and those with CFSPID to the need of adhering to a balanced, individually tailored diet, supported by appropriate vitamin supplementation.

## 5. Summary

The present study limitation includes the year-round food consumption—which shows varied meals that depend on the season of the year—and the lack of division into groups, according to vitamin dose and vitamin agent type used (β-carotene or retinol). In the future, a study should be attempted with a higher number of patients, with an evaluation of the effects of supplemented anti-oxidative vitamins on the functions of particular organs. For the CF patients, preparation should be standardized. In addition, in all groups of children, the vitamin D supplementation regularity and its preparation type needs to be assessed, and their time of exposure to sunlight needs to be considered.

Among the advantages of this study is the fact that this is one of the few studies that has described the phenotype of children with CFSPID [24,49,50]. The obtained data suggest that in asymptomatic children with CFSPID, despite the lack of routine vitamin supplementation, we did not observe significant deficits in this area. Currently, there are no indications for routine vitamin supplementation, other than vitamin D in children with CFSPID.

A further development of the knowledge about the effects of antioxidant vitamins A, E, and C, and of the pleiotropic effect of vitamin D on the CFSPID children’s systems and on CF patients in general, may bring potential benefits and improve their quality of life.

## 6. Conclusions

Both the children with CF and those with CFSPID did not fully adhere to the dietary recommendations for vitamin supplies, but a significant vitamin deficiency (mainly of vitamins D and E) was only found in the group of children with CF. In addition to vitamin supplementation, cystic fibrosis patients’ vitamin D and E body stores may be affected by pancreatic exocrine function and by mutations in the CFTR gene.

In children with CFSPID and CF, due to non-adherence to the recommended energy intake and total dietary vitamin intake, more attention should be paid to the necessity of adhering to the developed recommendations. Due to the presence of mutations with variable penetrance in the CFSPID group and the possibility that the cystic fibrosis phenotype may be revealed in the future, these children require further clinical evaluation, with an assessment of the pancreas and liver function, and an assessment of fat-soluble vitamins.

There were no significant differences between the children with CF and children with CFSPID in the anthropometric parameters.

## Figures and Tables

**Table 1:** Nutritional status of the study groups.

	Obesity *n* (%)	Overweight *n* (%)	Normal Body Weight *n* (%)	Body Underweight *n* (%)	*p*
CFSPID	0	6 (21.4)	17 (60.7)	5 (17.9)	0.574
CF	0	4 (12.9)	18 (58.1)	9 (29.0)	
HC	1 (3.3%)	5 (16.7)	20 (66.7)	4 (13.3)	

**Table 2:** Vitamin A, D, E, and C status among children in the study groups.

Vitamins	Vitamin Levels	CFSPID	CF	HC	*p*
A	Normal range *n* (%)	28 (100)	28 (90.3)	29 (96.7)	0.125
	Deficient *n* (%)	0	3 (9.7)	1 (3.3)	
	Excess *n* (%)	0	0	0	
D	30–50 ng/mL *n* (%)	22 (78.6)	12 (38.7)	22 (73.3)	0.001
	30–20 ng/mL *n* (%)	2 (7.1)	14 (45.2)	4 (13.3)	
	<20 ng/mL *n* (%)	0	5 (16.1)	1 (3.3)	
	>50 ng/mL *n* (%)	4 (14.3)	0	3 (10.0)	
E	Normal range *n* (%)	25 (89.3)	27 (87.1)	27 (90.0)	0.330
	Deficient *n* (%)	0	2 (6.5)	0	
	Excess *n* (%)	3 (10.7)	2 (6.5)	3 (10.0)	
C	Normal range *n* (%)	26 (92.9)	25 (80.6)	26 (86.7)	0.675
	Deficient *n* (%)	1 (3.6)	2 (6.5)	1 (3.3)	
	Excess *n* (%)	1 (3.6)	4 (12.9)	3 (10.0)	

**Table 3:** Distribution of vitamin A, D, E, and C blood concentration values among different groups.

Vitamin	CFSPID		CF		Healthy Children		*p*	Post-Hoc
	Me	IQR	Me	IQR	Me	IQR		
A	436.7	370.0–496.3	409.4	320.1–514.1	408.8	351.4–509.1	0.669	
D	43.3	32.8–48.2	28.3	22.1–33.9	33.3	30.1–38.7	0.001	HC vs. CFSID *p* > 0.05 HC vs. CF *p* = 0.004 CFSPID vs. CF *p* = 0.001
E	12.2	10.4–13.3	7.6	6.3–13.0	12.2	10.4–14.7	0.004	HC vs. CFSID *p* > 0.05 HC vs. CF *p* = 0.003 CFSPID vs. CF *p* = 0.006
α-tocopherol/cholesterol ratio	8.2	7.0–10.0	6.9	5.5–9.12	-	-	0.038	
C	46.8	31.4–58.4	48.2	40.2–60.3	49.6	44.2–55.7	0.604	

Me—Median, IQR—Inter-Quartile Range.

**Table 4:** Distribution of the content of vitamins A, D, E, and C in diets of particular groups.

	CFSPID		CF		*p*
	Me	IQR	Me	IQR	
μg of retinol equivalent/person/24 h	844.8	564.4–1141.4	1114.3	681.1–1502.0	0.171
mg of α-tocopherol equivalent/person/24 h	6.4	4.4–7.8	13.5	9.5–22.2	0.001
μg of cholecalciferol/person/24 h	1.7	0.8–3.8	5.6	2.7–8.4	0.001
mg of vitamin C/person/24 h	89.8	62.3–129.4	91.1	57.7–136.8	0.882

Me—median; IQR—inter-quartile range.

**Table 5:** Correlation between vitamin intake (diet and supplementation) and blood vitamin concentration in the groups.

Vitamin	CFSPID		CF	
	*R*	*p*	*R*	*p*
A	0.1	0.7	0.1	0.3
E	0.1	0.4	0.1	0.6
C	0.1	0.5	0.3	0.1
D	0.3	0.05	0.3	0.1

**Table 6:** Adherence to recommendations for daily vitamin intake in CFSPID and CF groups.

	Vit. A	*p*	Vit. D	*p*	Vit. E	*p*	Vit. C	*p*
CFSPID *n* (%)	24 (85.7%)	0.122	3 (10.7%)	0.001	17 (60.7%)	0.283	25 (89.3%)	0.603
CF *n* (%)	18 (64.3%)		25 (89.3%)		13 (46.4%)		27 (96.4%)	

**Table 7:** The daily consumption of calories in the CFSPID and CF groups based on three-day food records.

	CFSPID		CF		*p*
	Me	IQR	Me	IQR	
Daily caloric intake	1051	780–1366	1487	825–2129	0.08
%RDA	105	83–112	101	92–107	0.96

## Data Availability

The statistical analysis and database used to support the findings of this study may be released upon application to the Medical University of Silesia, department of paediatrics, which can be contacted by the corresponding author.

## Appendix A

The scope of reference values:

vit. A [ng/mL]: <1st year of life 200–800; 1–4 years of life: 250–800; >4th year of life: 300–800.

vit. E [μg/mL]: <4th year of life 3.8–16.0; 4–12 years of life: 4.0–16.0; >12th year of life: 5.0–20.0.

vit. C 28–85 μmol/L

The scope of reference values of vitamin D-(25)(OH)D):

Deficit: 0–20 ng/mL; suboptimal concentration: >20–30 ng/mL; optimal concentration: >30–50 ng/mL; high concentration: >50–100 ng/mL; potentially toxic concentration: >100 ng/mL.

Elastase-1 in faeces-normal result: 200 μg/g, lower values were considered abnormal and indicative of pancreatic exocrine insufficiency.

**Table A1:** Vitamin A—recommended dietary allowance (RDA)*;* estimated average requirement (EAR); and adequate intake (AI) for polish healthy children.

μg of Retinol Equivalent/Person/24 h (RAE)			
Group Sex, Age (Years Old)	AI	RDA	EAR
Infants			
0–0.5	350		
0.5–1	350		
Children			
1–3		400	280
4–6		450	300
7–9		500	350
Boys			
10–12		600	450
13–15		900	630
16–18		900	630
Girls			
10–12		600	430
13–15		700	490
16–18		700	490

**Table A2:** Recommended Vitamin E Intake for Children (RDA).

Age	mg of α-Tocopherol Equivalent/Person/24 h
0–6 months	4 mg
7–12 months	5 mg
1–3 years old	6 mg
4–8 years old	7 mg
9–13 years old	11 mg
14–18 years old	15 mg

**Table A3:** Vitamin D—adequate intake (AI) for Polish healthy children.

Group Age (Years Old)	μg of Cholecalciferol/Person/24 h
Infants	
0–0.5	10
0.5–1	10
Children	
1–3	15
4–6	15
7–9	15
10–12	15
13–15	15
16–18	15

**Table A4:** Vitamin C—recommended dietary allowance (RDA); estimated average requirement (EAR), and adequate intake (AI) for Polish healthy children.

Group Sex, Age (Years Old)	mg of Vitamin C/Person/24 h		
	AI	RDA	EAR
Infants			
0–0.5	20		
0.5–1	20		
Children			
1–3		40	30
4–6		50	40
7–9		50	40
Boys			
10–12		50	40
13–15		75	65
16–18		75	65
Girls			
10–12		50	40
13–15		65	55
16–18		65	55
